# Novel Property Cytotoxicity and Mechanism of Food Preservative Brevilaterins against Human Gastric Cancer Cells

**DOI:** 10.3390/foods12081732

**Published:** 2023-04-21

**Authors:** Zhou Chen, Dan Hong, Siting Li, Yingmin Jia

**Affiliations:** School of Food and Health, Beijing Technology and Business University, Beijing 100048, China

**Keywords:** food preservative, antimicrobial peptide, cytotoxicity, apoptosis, gastric cancer cells, Brevilaterins

## Abstract

Brevilaterins, antimicrobial peptides produced by *Brevibacillus laterosporus*, are regarded as excellent food preservatives and are popular as antimicrobial applications. Recent research has uncovered their potent cytotoxic effects against diverse cancer cells, thereby underscoring the pressing need for more extensive and intensive investigations into this use. In this study, we explored their novel function in inducing cytotoxicity to cancer cells and systematically investigated the mechanism of action of Brevilaterin B/C (BB/BC) in vivo. Proliferation, membrane permeability, and apoptotic rate were evaluated using CCK-8 assay, LDH assay, and Annexin V-FITC/PI kits. ROS levels and mitochondrial membrane potential were detected using the fluorescent probe DCFH-DA and JC-1. Our results demonstrated that both BB and BC at concentrations of 4–6 µg/mL significantly inhibited the proliferation and migration of gastric cancer cells BGC-823. Treatment with 4 µg/mL of BB/BC rapidly increased LDH levels in the supernatant of BGC-823 cells, leading to further investigation of the mechanism of apoptosis. We found that the apoptotic rate of BGC-823 cells significantly increased upon treatment with BB/BC, demonstrating their potent induction of apoptosis. BB/BC-induced ROS production in BGC-823 cells impaired their growth and induced apoptosis, indicating a close association between apoptosis and ROS elevation. Additionally, JC-1 aggregates rapidly accumulated after treatment with 4 µg/mL of BB/BC, suggesting changes in mitochondrial membrane potential and early apoptosis. Taken together, our findings revealed that BB and BC exhibit significant anticancer effects against gastric cancer cells, highlighting the promising potential of Brevilaterins as anticancer agents.

## 1. Introduction

Cancer is a leading cause of human mortality, with gastric cancer ranking among the top five fatal cancers [1]. Despite recent advances in diagnosis and treatment, complete surgical resection remains the sole approach for early-stage gastric cancer management [2]. However, the five-year survival rate post-surgery remains low at approximately 20%, highlighting significant safety concerns [3]. Therefore, a multimodal approach combining radiotherapy, chemotherapy, novel targeted drugs, or a combination of these has emerged as a means of enhancing the efficacy of gastric cancer treatment. The search for a novel, safe, and effective gastric cancer therapeutic agent or adjuvant has gradually become a primary approach to adjuvant therapy. Studies have revealed that antimicrobial peptides (AMPs) constitute a crucial element of the innate immune defense system with selective inhibitory effects on specific tumor cells, offering renewed hope for the development of potent and innovative anticancer drugs [4,5].

AMPs, commonly used as food preservatives, and also known as Bacteriocins, are a diverse group of peptides secreted by bacteria with the primary function of inhibiting pathogenic microorganisms. These cationic peptides achieve their bactericidal effect by binding to the microbial cell membrane [6]. Recent research has demonstrated that AMPs can also bind to the negatively charged outer membranes of cancer cells, resulting in cytotoxicity towards cancer cells through electrostatic interactions [7,8]. Thus, AMPs have emerged as a promising research area for the development of novel anticancer agents. Some AMPs derived from bacteria, such as Surfactin secreted by *Bacillus subtilis*, have been proven to have anticancer effects, exhibiting potent inhibitory activity against human breast cancer cell lines MCF-7 and MDA-MB-231, the leukemia cell line K562, the cervical cancer cell line HeLa, the rectal cancer cell line LoVo, the histiocytic lymphoma cell line U-937, and another mouse monocyte macrophage leukemia cell line RAW264 [7,9,10]. *Brevibacillus laterosporus* is a promising biocontrol bacterium that has garnered attention for its ability to produce a diverse range of antimicrobial peptides (AMPs) with potent broad-spectrum cytotoxicity against cancer cells. Recent studies have shed light on the potential therapeutic properties of these AMPs, including Bogorol B-JX, which has been shown to inhibit the proliferation of human histiocytic lymphoma cell lines and ConA-activated spleen cells [11], and Spergualin, which has demonstrated promising antitumor activity against transplantable leukemias in mice [12]. These findings highlight the potential of *Brevibacillus laterosporus* and its AMPs as a novel source of anticancer agents for future development. In recent years, our research team has been devoted to investigating the biological activity of Brevilaterins, a group of AMPs derived from *Br. laterosporus*. Our initial research showed that Brevilaterins could serve as excellent food preservatives, exhibiting broad-spectrum antibacterial activity against 50 strains of standard bacteria belonging to 43 species and 25 genera. These bacteria cover the majority of representative food-borne pathogens and spoilage bacteria commonly found in different foods [13]. Furthermore, our previous investigations have also revealed that Brevilaterins have the potential to effectively inhibit the proliferation of 27 human cancer cell lines, with a particularly strong inhibitory effect against gastric cancer cells [14,15]. However, the detailed anticancer characteristics and mechanisms of Brevilaterins against gastric cancer cells remain unclear and warrant further investigation. This could provide valuable insights into the development of novel therapeutic approaches for the treatment of gastric cancer. Thus, the present study is centered on Brevilaterins as the research subject, and aims to investigate its cytotoxic properties and mechanisms against BGC-823 gastric cancer cells in a comprehensive manner. The ultimate goal is to establish a theoretical basis for developing Brevilaterins as a potential anti-cancer drug, which could improve the treatment of gastric cancer. The insights gained from this study are expected to pave the way for the development of novel therapeutic strategies for effectively managing this debilitating disease.

## 2. Materials and Methods

### 2.1. AMPs, Cell Lines and Materials

Brevilaterin B (BB) and Brevilaterin C (BC) with a purity above 93% were prepared as per the methodology outlined in our previous publication [6]. The cell lines tested in this study included BGC-823 gastric cancer cells and its corresponding gastric epithelial cell GES-1, which is a human normal cell. BGC-823 and GES-1 cell lines were cultured using RPMI 1640 medium supplemented with 10% FBS and 1% penicillin-streptomycin cocktail, and cultured at 37 °C in a humidified atmosphere containing 5% CO_2_, as described previously [14]. FBS, penicillin, and cultural media were all purchased from Gibco (Oklahoma, ME, USA). The CCK-8 Assay Kit was procured from Biodee (Beijing, China), while the LDH and ROS Assay Kit were sourced from Jiancheng Bioengineering Institute (Nanjing, China). Additionally, the Annexin V/PI Staining Kit was purchased from BD biosciences (San Jose, CA, USA).

### 2.2. Effect of Brevilaterins on the Proliferation of Cells

Cell viability was assessed using the CCK-8 Assay Kit, following the previously described protocol [14]. Both BGC-823 and GES-1 cells were in the logarithmic growth phase, with a concentration of 5 × 10^4^ cells/mL. They were exposed to varying concentrations of Brevilaterins (0, 1, 2, 4, 8, 16, 32 µg/mL) for a duration of 48 h, followed by treatment with the CCK-8 solution and incubated for 4 h. The resulting cell viability was calculated using the following formula:

Cell viability (%) = (A2 − A0)/(A1 − A0) × 100%

A0: The absorption of the cultural medium.

A1: The absorption of the cells without treatment with Brevilaterin.

A2: The absorption of the cells treated with Brevilaterin.

### 2.3. Effect of Brevilaterins on the Migration of Cells

The Scratch method was employed to evaluate cell migration in a 24-well plate. To create the scratch, a culture-insert was placed in each well, and 70 µL of logarithmic cells (2 × 10^5^ cells/mL) were added. After 24 h of incubation, the supernatant was removed, and the culture-insert was carefully removed. The wells were then washed with PBS (1×) buffer and treated with varying concentrations of Brevilaterins (0, 4, 6 µg/mL) for 0, 24, and 48 h, respectively. Cell migration was assessed by measuring the Scratch healing rate, which was calculated using the following formula:

Scratch healing rate (%) = (W0 − W1)/W0 × 100%

W0: Original scratch width of cells without treatment with Brevilaterin.

W1: Final scratch width of cells treated with Brevilaterin.

### 2.4. Effect of Brevilaterins on the Membrane Permeability

To investigate membrane permeability, the levels of LDH released from cells were measured. Logarithmic cells (1 × 10^5^ cells/mL) were cultured for 24 h and subsequently treated with varying concentrations of Brevilaterins (0, 4, 6, 8 µg/mL) for an additional 24 h. Following treatment, the amount of LDH released into the supernatant of the treated cells was measured using an LDH Assay Kit.

### 2.5. Impact of Brevilaterins on the Ultrastructural Features of Cancer Cells

BGC-823 gastric cancer cells in the logarithmic growth phase were cultured at a density of 3 × 10^5^ cells/mL in a 10 cm cell culture dish containing 10 mL of medium for 24 h to allow adhesion. Subsequently, varying concentrations of antibacterial peptides (0 and 6 μg/mL) were added, and after 24 h, the cells were washed twice with PBS and treated with trypsin for 2 min. Complete culture medium was added, and the cells were transferred to a centrifuge tube, washed with PBS, and fixed with 2.5% electron microscopy fixation solution for 24 h at 4 °C. Ultrathin sections of the cells were prepared by dehydrating them with ethanol and acetone, embedding them in epoxy resin, and staining them with uranyl acetate and lead citrate for contrast. Transmission electron microscopy was used to visualize the ultrastructure of the cells.

### 2.6. Effect of Brevilaterins on the Apoptosis of Cancer Cells

To assess apoptosis in BGC-823, we measured apoptotic rate, changes in ROS levels, and mitochondrial membrane potential using the Annexin V-FITC/PI Kit, DCFH-DA, and JC-1 fluorescent probes, respectively. The methods employed were previously described by Chen et al. [14]. Specifically, logarithmically growing cells (2 × 10^5^ cells/mL) were cultured for 24 h, and then treated with various concentrations of Brevilaterins (0, 4, 6, 8 µg/mL) for an additional 24 h. To determine apoptotic rate, 100 µL of treated cells were mixed with 5 µL of Annexin V-FITC for 10 min, followed by the addition of 5 µL PI. The final mixture was diluted with 400 µL Binding buffer and quantitatively analyzed using a BD C6 flow cytometer. To measure ROS levels, treated cells were incubated with DCFH-DA for 20 min, washed twice with PBS (1×) buffer, harvested by trypsin digestion, and analyzed using a BD C6 flow cytometer. Finally, mitochondrial membrane potential was detected using the JC-1 fluorescent probe. Specifically, 500 µL of JC-1 and treated cells were mixed and incubated for 15 min. The cells were then harvested after trypsin digestion and analyzed using a BD C6 flow cytometer.

### 2.7. Statistical Analysis

Data were expressed as mean ± standard deviation and analyzed using one-way analysis of variance (ANOVA) in SPSS version 10.0 for Windows (SPSS Inc., Chicago, IL, USA) to determine any significant differences. The significant differences (*p* < 0.05) were observed and further analyzed using the least significant difference multiple-range test.

## 3. Results and Discussion

### 3.1. Inhibition of BB/BC on the Proliferation of Gastric Cancer Cells

**Figure 1:**
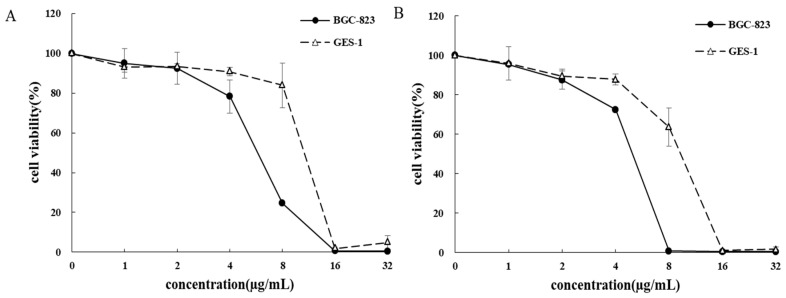
Effect of BB (**A**) and BC (**B**) on the proliferation of gastric cancer cells BGC-823 and gastric epithelial cells GES-1.

**Figure 2:**
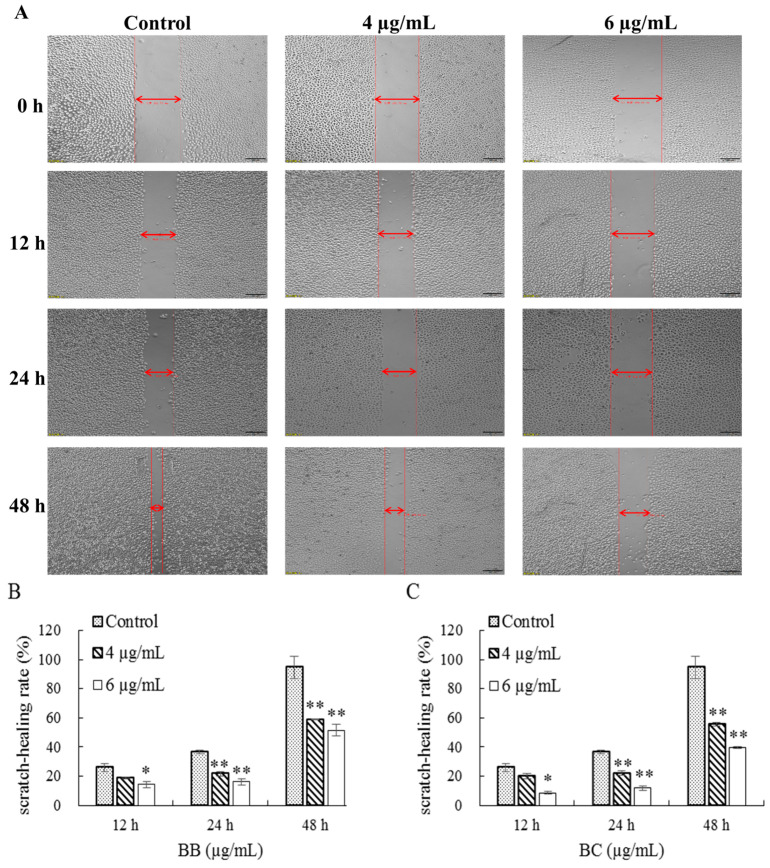
Effect of BB and BC on the inhibition of migration of cancer cell BGC-823: (**A**) the morphology of BGC-823 cells after treatment by BB; (**B**,**C**) the scratch-healing rates of BGC-823 cells after treatment by BB and BC, respectively. The red twin-arrows indicate cell scratch’s length. The data are expressed as the means ± SEs (* *p* < 0.05; ** *p* < 0.01).

In this study, we aimed to investigate the impact of BB/BC, a spectrum anticancer peptide [14,15], on gastric cancer cells. Our results showed that BB/BC strongly inhibited cell proliferation in a dose-dependent manner (Figure 1). Specifically, as the concentration increased from 2.0 µg/mL to 16.0 µg/mL, the viability of gastric cancer cells BGC-823 significantly decreased, whereas the corresponding gastric epithelial cells GES-1 were only slightly affected. Notably, 8.0 µg/mL of BB/BC strongly inhibited the viability of cancer cells BGC-823 while having minimal effect on its corresponding human normal gastric epithelial cells, GES-1, suggesting their potential for selective inhibition of human cells. Moreover, the inhibitory effects of BB/BC on BGC-823 were further elucidated through migration assays (Figure 2). Compared to the control group, the antimicrobial peptides BB and BC exhibited a concentration-dependent enhancement in their inhibition effect on cell migration, leading to a decreased rate of cell scratch closure during the identical treatment duration. Specifically, treatment with 4.0 µg/mL and 6.0 µg/mL of BB for 48 h resulted in significantly decreased scratch-healing rates of BGC-823, at 58.91% and 51.53%, respectively, in contrast to the control group’s rate of 94.57% (Figure 2B). Similarly, treatment with BC also showed a similar trend with scratch-healing rates of 55.45% and 39.46%, respectively (Figure 2C).

In recent years, the potential of AMPs as a source of effective cancer treatment has garnered significant attention. Originally identified as a component of the human innate defense system, AMPs have been extensively investigated for their therapeutic applications against various diseases, including cancer. Numerous studies have demonstrated that AMPs exhibit potent anticancer properties that surpass those of currently available therapies, such as the AMP named Bogorol B-JX that is secreted by the strain of *Br. laterosporus*, which efficiently inhibits proliferation of the human histiocytic lymphoma cell line U-937 [11,16,17,18]. Our previous research has also identified Brevilaterins, produced by *Br. laterosporus* S62-9, as a promising new class of molecules with potent anticancer activity. Indeed, Brevilaterins could display variable inhibition towards 27 cancer cells in a dose-dependent manner [14,15]. In this study, we specifically focused on the inhibitory effects of Brevilaterins on gastric cancer cells and described their properties as potential anticancer agents. This marks the first report that Brevilaterins demonstrate activity against gastric cancer cells, thereby further underscoring their potential as a novel class of AMPs for cancer therapy.

### 3.2. Effect on the Changes in Gastric Cancer Cell Morphology of BB/BC

**Figure 3:**
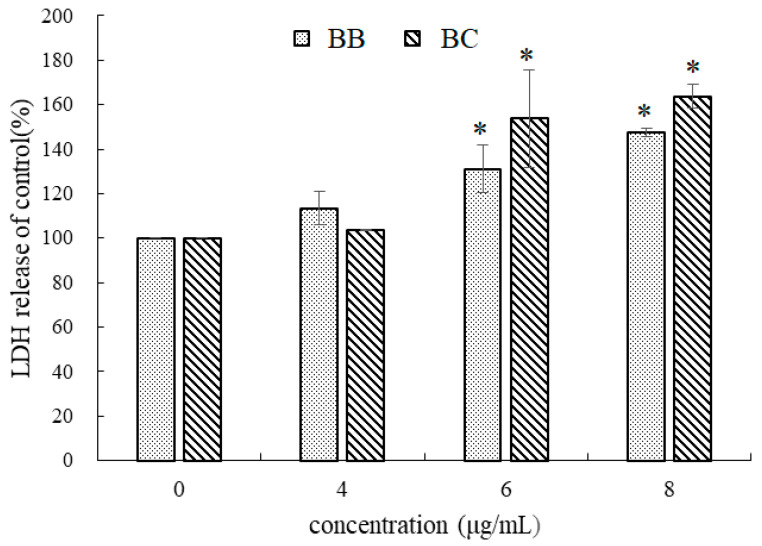
Changes of LDH release rate in BGC-823 cells after treatment by BB and BC (* *p* < 0.05).

In comparison to the control group that was not treated with antimicrobial peptides (AMPs), both BB and BC demonstrated a significant increase in LDH levels in BGC-823 cells (Figure 3). The LDH release rate in BGC-823 cells increased from 113.41% to 153.78% as the concentration of BB increased from 4 µg/mL to 8 µg/mL. Similarly, the LDH release rate in BGC-823 cells increased from 103.68% to 163.8% at the same concentrations of BC. These results indicate that BC is more effective than BB in damaging the cell membrane of gastric cancer cells BGC-823, which may be due to their differences in their amino acid sequence and molecular structure. In instances of cell apoptosis or necrosis, various cellular mechanisms are disrupted, leading to changes in the structural and functional integrity of the cell membrane. As a consequence, the cell membrane may become compromised, resulting in the release of intracellular contents, such as enzymes and other cytoplasmic proteins, into the extracellular environment. This phenomenon is often associated with an increase in the activity of certain cellular enzymes, such as LDH, which is released into the culture medium and serves as a typical marker of cell membrane damage and the extent of cell death [19,20]. Herein, the LDH release from BGC-823 cells confirms that both BB and BC induce apoptosis, leading to cell death. This precise feedback was also observed in human promyelocytic leukemia HL-60 cells after treatment with the AMP cecropin A [21].

**Figure 4:**
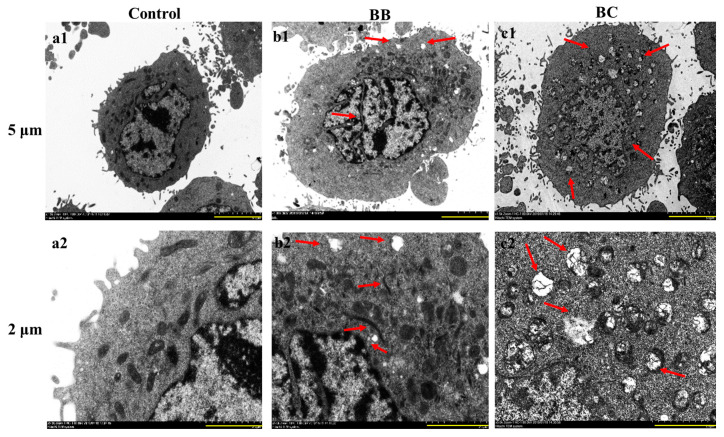
Effects of BB and BC on the ultrastructure in BGC-823: (**a1**,**a2**) BGC-823 cells without treatment with Brevilaterins; (**b1**,**b2**) BGC-823 cells after treatment by BB for 24 h; (**c1**,**c2**) BGC-823 cells after treatment by BC for 24 h. The arrows indicate typical changes, such as the appearance of autophagic vacuoles and nuclear membrane “folding” within the cells. The yellow lines represent a scale of corresponding size.

Morphological alterations are the cornerstone for identifying apoptosis and represent the most straightforward approach, featuring chromatin condensation, fragmented DNA, swollen mitochondria, or the emergence of apoptotic bodies. The cellular ultrastructure can provide a more detailed observation of the fine changes in cell structure following AMP treatment, thus corroborating the influence of AMPs on cell morphology. Observation of the effects of BB and BC on the ultrastructure of BGC-823 cells was conducted using transmission electron microscopy (TEM) (Figure 4). BGC-823 cells without treatment displayed abundant and intact organelles, evenly distributed chromosomes in the nucleus, and clear nucleoli. The overall cell morphology was intact, with a rich population of microvilli on the cell surface. However, after treatment with 6 µg/mL of BB, the number of microvilli on the cell surface decreased, and numerous autophagic vacuoles appeared inside the cells. The nuclear membrane displayed a “wrinkled” appearance, with heterochromatin in the nucleus increasing and forming chunky edges near the nuclear membrane. Some organelles exhibited a loss of their internal structure and assumed a “linear” configuration, displaying typical features of apoptosis. Treatment with 6 µg/mL of BC resulted in evident swelling of both the cell volume and nucleus of BGC-823 cells, with many vacuoles appearing. Internal structures such as mitochondrial cristae disappeared. These findings suggest that 6 µg/mL of BB and BC can modulate the ultrastructure of BGC-823 cells.

**Figure 5:**
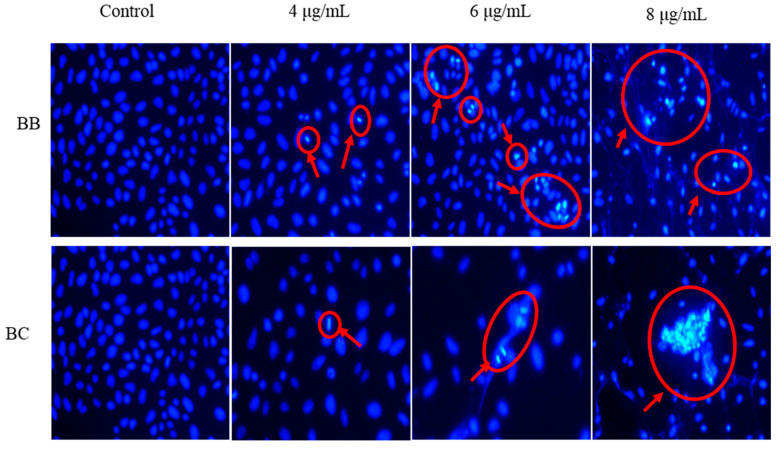
Effects of BB and BC on the nucleus in BGC-823 (enlargement factor: ×100; Circles: Phenomenon characterized by the condensation of the nucleus, leading to the dense staining and fragmentation of a portion of chromatin).

The cell nucleus, being a critical organelle in cells, plays a pivotal role in cell growth and metabolism. Upon the occurrence of cell apoptosis, notable changes in cell morphology ensue, including a reduction in cell volume, the aggregation of chromatin, and the formation of apoptotic bodies. To investigate whether the inhibitory effect of AMPs on BGC-823 cells is caused by cell apoptosis, Hoechst 33258 fluorescence––a blue fluorescent dye that can pass through the cell membrane, and commonly used for detecting cell apoptosis––staining was used to observe the morphological changes of the cell nucleus, as depicted in Figure 5. In the control group, chromatin is uniformly distributed, and the cell nuclei exhibits uniform light blue staining. However, exposure of BGC-823 cells to different concentrations of BB and BC results in nuclear condensation and fragmentation of chromatin, which are hallmarks of apoptosis. Moreover, as the concentration of BB and BC increases, there is a decrease in the overall number of cell nuclei, accompanied by a relative increase in the number of cells with densely stained chromatin. Notably, exposure to 6 µg/mL and 8 µg/mL of BC leads to a significant reduction in the overall number of cells compared to the control group, with most cell nuclei becoming deformed, fragmented, and clustering together. These changes in cell nuclei morphology clearly demonstrate that both BB and BC can induce apoptosis in BGC-823 cells, with BC being more potent in inducing apoptosis compared to BB.

### 3.3. Further Study on the Apoptotic Mechanism of BB/BC towards Gastric Cancer Cells

Building on previous research findings, we hypothesized that BB/BC induces apoptosis in gastric cancer cells. In this study, we aimed to investigate the mechanism behind this process by examining the apoptotic rate, ROS levels, and mitochondrial membrane potential as assessment indicators.

**Figure 6:**
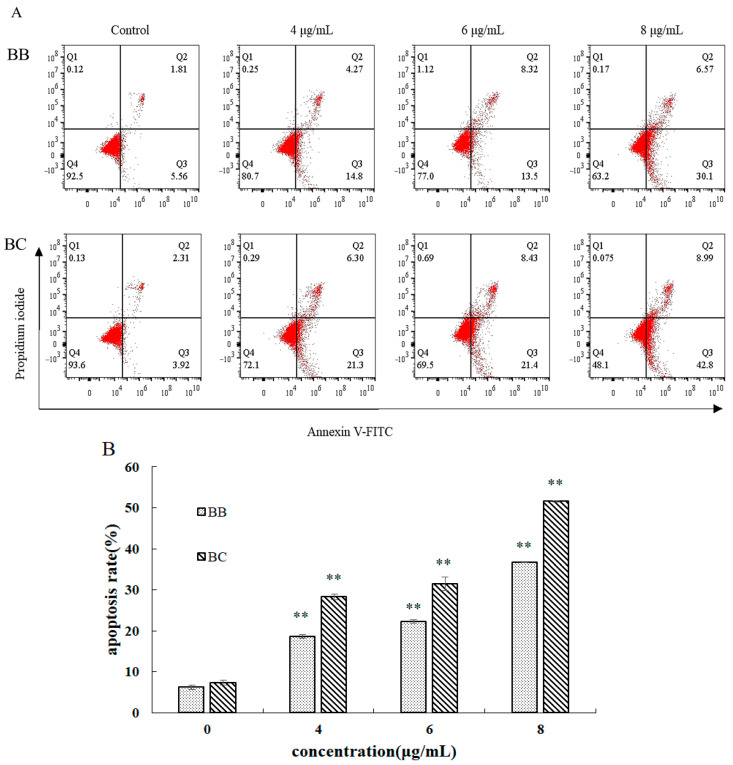
Effect of BB and BC on the apoptotic rate of BGC-823 cells ((**A**) FITC/PI double-staining flow chart; (**B**) statistical chart of apoptotic rate; ** *p* < 0.01).

As depicted in Figure 6A, treatment with BB/BC resulted in a gradual increase in apoptotic rates of BGC-823 cells. At concentrations of 4 µg/mL, 6 µg/mL, and 8 µg/mL of BB, approximately 18.68%, 22.25%, and 36.69% of cancer cells underwent apoptosis, respectively (Figure 6B). Similarly, treatment with BC at the same concentrations led to even higher apoptotic rates of BGC-823 cells, reaching 28.31%, 31.46%, and 51.61%, respectively, indicating that BC exhibited stronger inhibitory effects (Figure 6B). These findings suggest that BB and BC may induce apoptosis in BGC-823 cells, highlighting their potential as effective anticancer agents. Apoptosis is a crucial physiological process that regulates the balance between cell division and cell death to maintain cellular homeostasis and prevent uncontrolled proliferation of cancer cells [22]. During the early stages of cellular apoptosis, a critical event is the translocation of phosphatidylserine from the inner to the outer leaflet of the cell membrane. This phenomenon can be detected using Annexin V-FITC staining, which specifically binds to phosphatidylserine and produces green fluorescence in apoptotic cells. In contrast, necrotic or late-stage apoptotic cells can be identified using PI staining, which penetrates cells with compromised plasma membrane integrity and emits red fluorescence upon binding to nucleic acids. The relative distribution of green and red fluorescence provides essential parameters for quantifying the level of apoptosis in cells, allowing for a direct measurement of cell death. This approach has been widely employed in various studies and is considered a robust tool for monitoring apoptosis in vitro and in vivo. As evidenced by our previous research, BB/BC could induce apoptosis in epidermal carcinoma cells A431, with a remarkable apoptotic rate exceeding 70% [14,15]. Moreover, AMPs from various origins, including ranatuerin-2PLx and temporin-1CEa, were found to elevate the apoptotic rate during the induction of cell apoptosis in PC-3 M and Bcap-37 cell lines [23,24].

**Figure 7:**
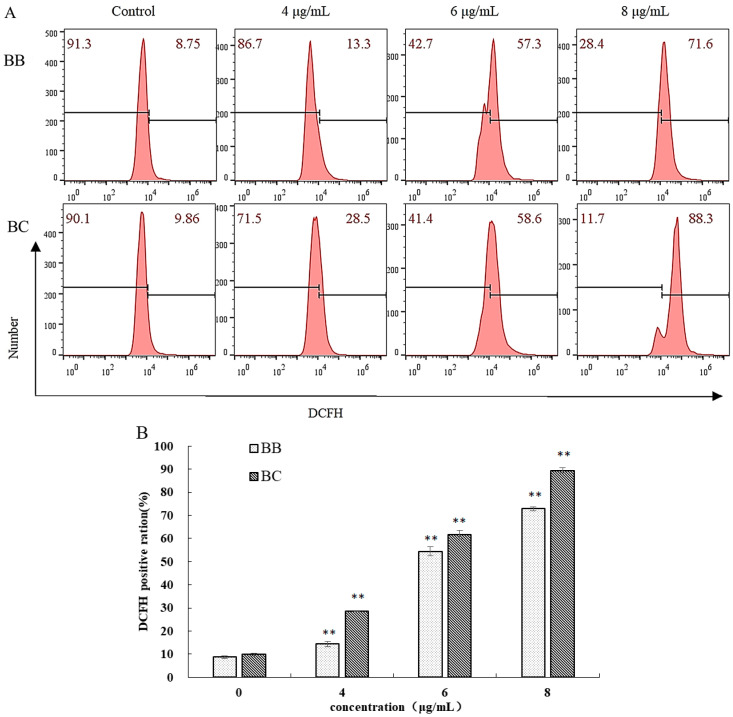
Effect of BB and BC on the ROS levels of BGC-823 cells ((**A**) DCFH-DA monochrome flow chart; (**B**) DCFH positive proportion statistical chart; ** *p* < 0.01).

A fluorescent probe DCFH-DA was utilized to quantify the levels of ROS, and the outcomes were presented in Figure 7. The detected levels of ROS in BGC-823 gastric cancer cells increased significantly in a dose-dependent manner following treatment with BB/BC. Specifically, the DCFH positive ratio of BGC-823 cells showed an increase, peaking at 14.28%, 54.35%, and 72.77% after 24 h of exposure to 4, 6, and 8 µg/mL of BB, respectively. Likewise, a dose-dependent increase in the DCFH positive ratio was also observed in the same cancer cells treated with BC, reaching 28.5%, 61.57%, and 89.38% after 24 h exposure to 4, 6, and 8 µg/mL of BC, respectively. Mitochondria serve as the primary energy source of cells and play a critical role in cellular growth, proliferation, and differentiation. In the field of cancer treatment, several anti-tumor drugs activate mitochondrial pathways to exert their anti-cancer effects, which frequently entail the production of ROS. During apoptosis, there is a significant accumulation of ROS, which can affect the metabolic activities of cells and cause characteristic morphological changes, as established in previous research [25,26,27]. Therefore, the evaluation of drug-induced cell damage can be effectively assessed, and treatment adjustments can be made in real-time by detecting ROS levels during the use of drugs to combat cancer cells [28]. Studies using AMPs to induce apoptosis in different cancer cells have consistently demonstrated this typical biological feature, as seen in BB/BC inducing apoptosis and causing an increase in ROS levels in epidermal carcinoma cells A431 [14,15]. Similarly, AMP such as Bogorol B-JX from *Br. Laterosporus* can cause ROS accumulation in U-937 cells [11], another pentapeptide can induce apoptosis in HL-60 cells and increase their intracellular ROS levels [29], and the AMP CM4 has the same effect on breast cancer cells MX-1, MCF-7, and MDA-MB-231 [30,31].

**Figure 8:**
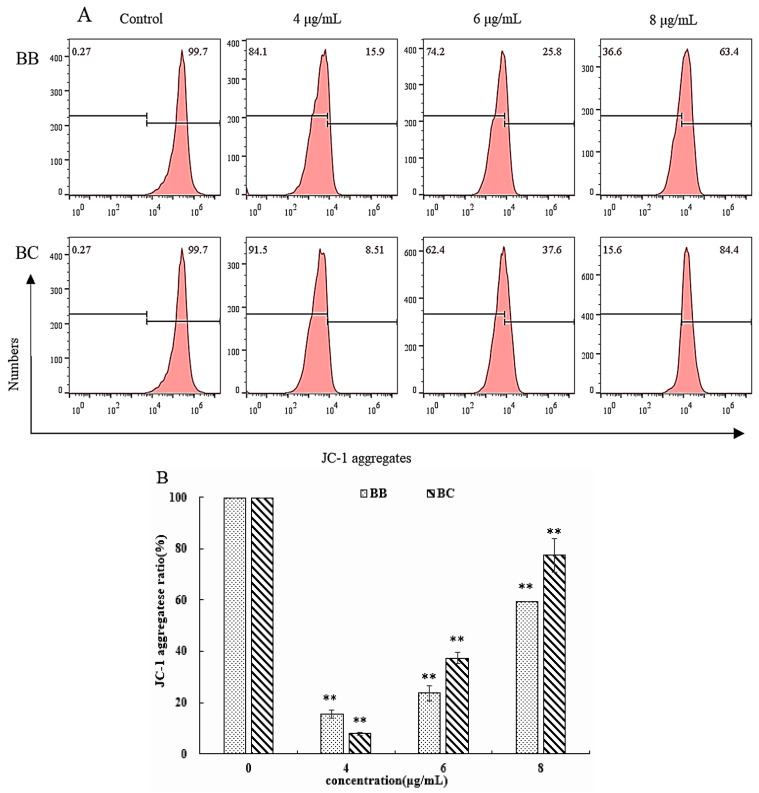
Effect of BB and BC on the mitochondrial membrane potential of BGC-823 cells ((**A**) JC-1 monochrome flow chart; (**B**) JC-1 positive aggregate statistical chart; ** *p* < 0.01).

To evaluate the mitochondrial membrane potential of BGC-823 cells, fluorescence probe JC-1, which exists in monomeric and aggregated forms, was used (Figure 8). Consistent with its effect on ROS levels, treatment with a concentration of 4 µg/mL of BB/BC effectively prevented the formation of JC-1 aggregates, indicating the induction of early-stage apoptosis in BGC-823 cells. However, with higher concentrations of BB/BC, the formation of JC-1 aggregates increased, accompanied by increased apoptotic rates and ROS levels.

Apoptosis is a complex cellular death process regulated by multiple factors that involves the participation of reactive oxygen species (ROS). ROS can function as an external inducer or an intermediate product of other stimuli that trigger apoptosis in cells. While ROS can be produced through various pathways, the mitochondria are the primary intracellular source of ROS generation, occurring within the mitochondrial electron transport chain [11]. Accumulation of intracellular ROS can disrupt the oxidative respiratory chain, alter the mitochondrial membrane potential, and ultimately result in mitochondrial dysfunction and apoptosis [25]. During apoptosis, the dissipation of mitochondrial membrane potential is a characteristic early signal of apoptosis. This process can be detected by the change in JC-1 fluorescence from red to green, which allows for the formation of JC-1 aggregates in the mitochondrial matrix and the quantification of mitochondrial membrane potential. The addition of BB/BC has been shown to effectively decrease the mitochondrial membrane potential in BGC-823 cells, indicating their ability to induce early apoptosis in these cancer cells. These findings are consistent with those observed in epidermal carcinoma cells A431 [14,15] and other AMPs such as Pardaxin and AMP B11, which have been shown to induce early apoptosis in cancer cells HT-1080 and Hela, respectively [31,32]. With increasing concentrations and duration of BB/BC treatment, an upward trend in mitochondrial membrane potential was observed in BGC-823 cells, which may be attributed to the fact that BB/BC triggers apoptosis in these cells from early to late stages at increasing concentrations [11].

## 4. Conclusions

In conclusion, this study investigated the efficacy of two newly discovered antimicrobial peptides (AMPs), BB and BC, against BGC-823 gastric cancer cells. The findings revealed that treatment with 4–6 µg/mL of BB/BC effectively hindered cell migration, altered the ultrastructure and nuclear morphology of cancer cells, and induced the release of a significant amount of LDH, evidencing their potential to induce apoptosis in BGC-823 cells. Further analysis unveiled that BB and BC both triggered a noteworthy accumulation of reactive oxygen species (ROS) in cancer cells and disturbed mitochondrial function. At lower concentrations, BB/BC induced early apoptosis in BGC-823 cells, whereas at higher concentrations, they fostered late-stage apoptosis. Building on the aforementioned findings, we have ascertained that BB and BC exert pronounced inhibitory effects on BGC-823 gastric cancer cells. This breakthrough discovery is poised to significantly advance the development and exploitation of Brevilaterins as a promising anticancer drug component, thereby earmarking it as a pivotal avenue for future research. Moving forward, our investigations into Brevilaterins will encompass diverse test hosts, including an in depth exploration of its stability within the host during utilization, as well as an evaluation of its toxicity potency against gastric cancer cells during in vivo application.

## Data Availability

No new data were created or analyzed in this study. Data sharing is not applicable to this article.
